# Yeast Reporter Assay to Identify Cellular Components of Ricin Toxin A Chain Trafficking

**DOI:** 10.3390/toxins8120366

**Published:** 2016-12-06

**Authors:** Björn Becker, Tina Schnöder, Manfred J. Schmitt

**Affiliations:** Molecular and Cell Biology, Department of Biosciences and Center of Human and Molecular Biology (ZHMB), Saarland University, Saarbrücken D-66123, Germany; b.becker@microbiol.uni-sb.de (B.B.); Tina.Schnoeder@med.uni-jena.de (T.S.)

**Keywords:** *S. cerevisiae*, ricin toxin A chain (RTA), ribosome inactivating protein (RIP), retrograde protein transport, trans-Golgi network (TGN), endoplasmic reticulum (ER)

## Abstract

RTA, the catalytic A-subunit of the ribosome inactivating A/B toxin ricin, inhibits eukaryotic protein biosynthesis by depurination of 28S rRNA. Although cell surface binding of ricin holotoxin is mainly mediated through its B-subunit (RTB), sole application of RTA is also toxic, albeit to a significantly lower extent, suggesting alternative pathways for toxin uptake and transport. Since ricin toxin trafficking in mammalian cells is still not fully understood, we developed a GFP-based reporter assay in yeast that allows rapid identification of cellular components required for RTA uptake and subsequent transport through a target cell. We hereby show that Ypt6p, Sft2p and GARP-complex components play an important role in RTA transport, while neither the retromer complex nor COPIB vesicles are part of the transport machinery. Analyses of yeast knock-out mutants with chromosomal deletion in genes whose products regulate ADP-ribosylation factor GTPases (Arf-GTPases) and/or retrograde Golgi-to-ER (endoplasmic reticulum) transport identified Sso1p, Snc1p, Rer1p, Sec22p, Erv46p, Gea1p and Glo3p as novel components in RTA transport, suggesting the developed reporter assay as a powerful tool to dissect the multistep processes of host cell intoxication in yeast.

## 1. Introduction

Understanding intracellular ricin transport is important for the development of effective strategies against acute ricin intoxication. As a member of the A/B toxin family, ricin is a highly potent protein toxin from the seeds of the castor oil plant *Ricinus communis* that belongs to class-II ribosome inactivating proteins (RIPs) [1]. It consists of two disulfide-bonded polypeptides, amongst which the B-chain (RTB) serves as the cell binding subunit mediating toxin uptake by mammalian target cells [2]. The cytotoxic A-chain of ricin (RTA) acts as *N*-glycosidase that cleaves a specific adenine residue within a conserved sarcin/ricin loop of eukaryotic 28S rRNA, which subsequently causes a block in eEF-2 mediated translation initiation followed by rapid cell death [3].

Over the years, intensive analyses of ricin trafficking in mammalian cells identified a complex network of pathways that is parasitized by the toxin [4,5,6,7,8,9]. Although intoxication initiates by RTB binding to terminal galactose and/or *N*-acetylglucosamine residues in cell surface proteins or lipids, followed by ricin uptake through clathrin-dependent as well as -independent endocytosis and vesicular transport to early endosomes, RTA without RTB is likewise capable to kill yeast and mammalian cells (IC_50_ of 50–100 µg/mL), though cell killing is much more efficient for ricin holotoxin (IC_50_ of 2 ng/mL) [10,11,12,13,14,15,16,17]. Whilst most of the dimeric toxin is subsequently exocytosed and/or degraded in lysosomes, only 5% of the internalized toxin reaches the trans-Golgi network (TGN) [18] through retrograde transport by components such as the GARP-complex [5], Stx5 [6] and the GTPases Rab6A and Rab6A′ [7], while neither retromer components [5] nor the GTPases Rab9 and Rab11 [11] are required for endosome–TGN transport. Retrograde toxin transport within the Golgi is mediated by the TRAPP-complex [5] followed by backward transport to the endoplasmic reticulum (ER). Since ricin itself does not contain an ER retention/targeting signal that could potentially mediate its interaction with KDEL receptors of the target cell, it has been proposed that RTB binds to resident luminal ER proteins, which indirectly allows toxin transport to the ER [19,20]. Within the ER, ricin is recognized by EDEM1 and EDEM2 [21,22] and subsequently retrotranslocates into the cytosol, most likely through the Sec61 translocon by using components of the ER-associated protein degradation (ERAD) machinery, including Derlins 1–3 [23]. Consequently, inhibition of retrograde transport, either by chemical inhibitors or via specific antibodies, efficiently protects mice and cell lines against ricin intoxication, indicating that detailed knowledge and understanding of intracellular toxin trafficking is a prerequisite for the development of a protective ricin antidote which, until now, is still not available [6,24,25].

Interestingly, intact yeast cells are phenotypically ricin resistant due to a lack of galactosylated RTB binding sites at their cell surface, while the same cells become ricin sensitive after RTA expression in the ER [23,26,27] or when exogenously applied to cell wall lacking spheroplasts [17]. This important observation turns yeast into an attractive model to study RTA uptake and intracellular transport in a lower eukaryote. Furthermore, as retrograde protein transport in its basic mechanisms is similar between yeast and mammalian cells (for reviews see e.g., [28,29,30,31]), we focused on the development of a yeast-based assay to identify cellular components of intracellular RTA transport and to address three major questions: (i) Which proteins are involved in RTA transport from the plasma membrane through the endosomal compartment to the ER? (ii) Are there any similarities and/or differences in RTA trafficking between yeast and mammalian cells? (iii) How useful is yeast as a model to analyze ricin transport pathways?

## 2. Results and Discussion

### 2.1. Fluorescence-Based Reporter Assay for RTA Toxicity in Yeast

**Figure 1:**
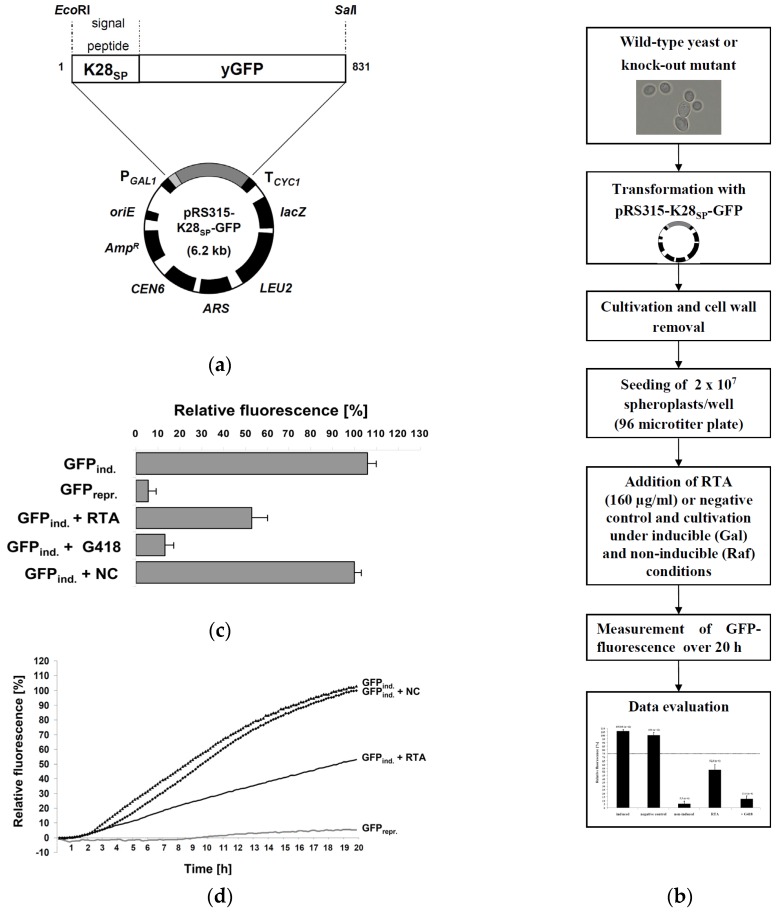
(**a**) Schematic overview of the reporter plasmid used to screen yeast for proteins involved in ricin toxin A chain (RTA) transport. Yeast enhanced GFP extended by an *N*-terminal signal peptide (SP) derived from K28 preprotoxin [34] is placed under transcriptional control of the *GAL1* promoter and *CYC1* terminator, allowing inducible expression in the presence of galactose. Indicated numbers correspond to amino acid position in GFP; (**b**) Experimental assay setup. After transformation of a particular yeast strain with pRS315-K28_SP_-GFP and cell wall removal by zymolyase treatment, 2 × 10^7^ spheroplasts were seeded in 96 microtiter plates and GFP expression was induced by the addition of 3% galactose. Simultaneously, RTA or the negative control sample was added and fluorescence development was measured over a time window of 20 h; (**c**) Relative fluorescence emission of 2 × 10^7^ wild-type yeast spheroplasts expressing GFP from the reporter plasmid pRS315-K28_SS_-GFP under induced (3% galactose, GFP_ind._) and non-induced (2% raffinose, GFP_repr._) culture conditions. Yeast spheroplasts were also incubated in the presence of RTA (5 µM), G418 (300 µg/mL) or the negative control (NC) over 20 h. Standard deviation is indicated; all measurements were repeated 6 to 12 times as independent experiments without technical replicates; (**d**) Time course of GFP fluorescence development of wild-type yeast spheroplasts expressing GFP from the reporter plasmid pRS315-K28_SS_-GFP in the presence (RTA) or absence (NC) of 5 µM RTA.

To screen yeast for cellular components involved in retrograde toxin transport after external RTA application, a GFP-based reporter assay was designed in which RTA toxicity is measured indirectly through fluorescence emission after in vivo translation of a secreted GFP reporter containing an *N*-terminal signal peptide and ER import signal derived from the yeast viral K28 preprotoxin [32]. Expression of this reporter (K28SP-GFP) is driven from the inducible *GAL1* promoter and requires active protein biogenesis for fluorescence emission. Since RTA efficiently blocks mRNA translation by depurinating 28S rRNA [19,33], this assay is expected to allow the identification of both known and novel host cell proteins that are involved in intracellular RTA transport. In case that RTA uptake and/or compartmental transport is negatively affected in a particular yeast knock-out mutant, this should be in line with an increased in vivo fluorescence of the reporter. The general setup of this bioassay and its use in an *S. cerevisiae* wild-type strain and various isogenic knock-out mutants after transformation with the reporter plasmid is outlined in Figure 1a. To bypass the lack of specific RTA binding sites at the yeast cell surface, spheroplasts of yeast transformants are cultivated in microtiter plates under conditions of induced GFP expression in the presence or absence of externally applied RTA and fluorescence kinetics is measured in wild-type and mutant spheroplasts over a time window of 20 h (Figure 1b).

In a first experiment, the effect of extracellular applied RTA on wild-type spheroplasts was analyzed; a Ni^2+^/NTA-purified culture supernatant from *E. coli* carrying the empty vector was included as negative control, whereas cells treated with the aminoglycoside antibiotic and protein biosynthesis blocker geneticin (G418) served as positive control. As expected, G418-treated cells revealed only a slight but clearly detectable increase in GFP fluorescence after induction (13.4% ± 3.9%) while no fluorescence (5.5% ± 3.6%) was seen in non-induced cells (Figure 1c). Induced cells (105.9% ± 4.2%) showed a similar level in fluorescence as negative control cells (set to 100%), while fluorescence significantly declined in RTA-treated spheroplasts (52.8% ± 7.2%). Based on the cytotoxic effect of RTA against yeast, an IC_50_ of approximately 5 µM (160 µg/mL) can be calculated, which is remarkably close to the reported toxicity of RTA against mammalian cells with IC_50_ values ranging from 1.5 to 3 µM (50–100 µg/mL), while toxicity of ricin holotoxin, consisting of RTA and RTB, is several orders of magnitude higher with an IC_50_ of 30 pM (2 ng/mL) [16]. Thus, RTA seems two to three times less toxic against yeast in comparison to mammalian cells and approximately 10^5^ times less toxic than ricin holotoxin (for which IC_50_ data are not available in yeast).

To get an idea about the timing of GFP expression and its translational inhibition by RTA in our experimental setup, relative GFP fluorescence was determined over a time period of 20 h. As illustrated in Figure 1d, GFP fluorescence became detectable and continuously increased 90 min after *GAL1-*induced expression, while the inhibitory effect of RTA on GFP translation and fluorescence became detectable after 210 min; this delay in RTA-mediated fluorescence decrease most likely reflects the kinetics of RTA uptake and intracellular transport to the cytosol. Although the simultaneous induction of GFP expression and RTA application caused an increase in the GFP background signal, the chosen 20 h end-point GFP signal nevertheless turned out suitable to detect significant differences in fluorescence inhibition between toxin-treated and control cells. In this way, a stringent correlation between in vivo reporter fluorescence and the level of protein biosynthesis after external RTA application could be demonstrated, confirming the suitability of this assay to detect the impact of RTA on in vivo protein biosynthesis in the genetic background of different knock-out mutants.

### 2.2. Assay Validation

**Figure 2:**
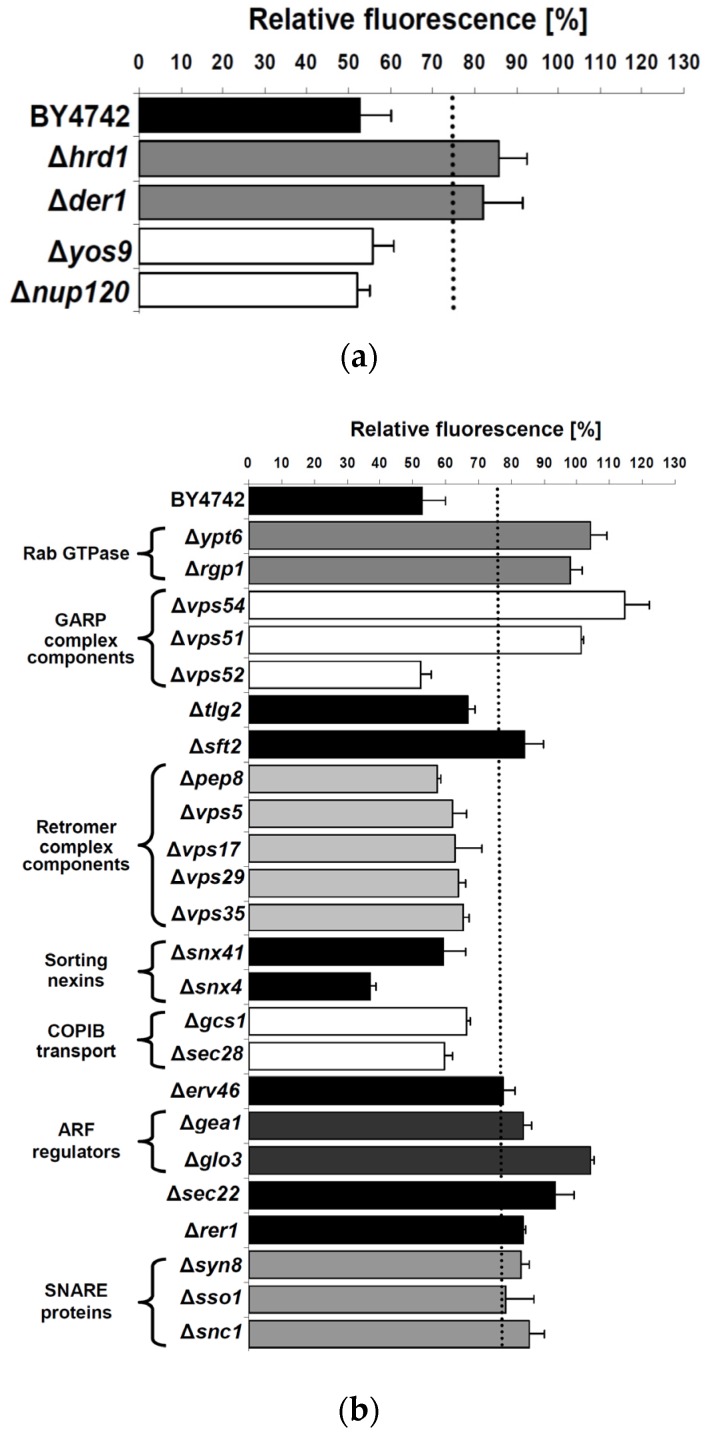
(**a**) Assay validation. Relative fluorescence of *∆der*1, *∆hrd*1, *∆yos*9 and *∆nup*120 knock-out mutant spheroplasts is indicated. Measurements were performed in the presence of RTA (5 µM) under induced culture conditions (3% galactose) over 20 h. Shown fluorescence values at 20 h were normalized to the fluorescence of spheroplasts treated with the negative control sample; (**b**) Impact of the indicated yeast deletion mutants on RTA trafficking. Fluorescence values of 2 × 10^7^ yeast spheroplasts of each mutant were displayed after 20 h induction (3% galactose) in the presence of RTA (5 µM). Values shown in (**a**,**b**) were normalized to the fluorescence of negative control spheroplasts, mean values and standard deviations are indicated. All measurements were at least repeated 3 to 12 times as independent experiments without technical replicates. The dotted line in (**a**) and (**b**) indicates the chosen threshold of 75% for a positive hit.

To validate the yeast bioassay, the influence of external RTA application was determined in yeast mutants with chromosomal deletions in genes whose products are known to either affect (*Δder*1, *Δhrd*1) or not affect (*Δyos*9, *Δnup*120) RTA in vivo toxicity. In the case of ERAD components, Hrd1p and Der1p have already been demonstrated to be involved in ER-to-cytosol retrotranslocation of RTA in yeast, while siRNA mediated knock-down of the mammalian Der1p homologue, Derlin1, causes reduced sensitivity against ricin [5,35]. Thus, both mutants should show an increased fluorescence in the yeast reporter assay. As negative controls, deletion mutants *Δyos*9 and *Δnup*120 were included since neither a lack of the ER quality control lectin Yos9p nor a lack in the nuclear pore protein Nup20p affect RTA transport or in vivo toxicity [35]. As shown in Figure 2a, GFP fluorescence in RTA treated *Δder*1 and *Δhrd*1 cells was significantly increased to 82.1% ± 9.3% and 85.7% ± 6.9%, while fluorescence in *Δyos*9 (55.7% ± 5%) and *Δnup*120 (52% ± 3.2%) spheroplasts did not differ from wild-type cells. In a final validation, a threshold of significance (dotted line in Figure 2a,b) based on fluorescence emission in the positive controls was set to 75% (in comparison to the 52.8% signal for the average fluorescence of RTA-treated wild-type cells) to exclude false positive hits and to identify only those knock-out mutants whose defects have a strong impact on RTA trafficking.

### 2.3. Cellular Pathways Involved in Endosome-to-Golgi Transport of RTA

Having confirmed the general suitability of the reporter assay, we first focused on cellular components such as Ypt6p, Rgp1p and Ric1p, which are known to play a central role in retrograde protein transport from endosomes to the TGN [36,37]. In particular, Ypt6p is involved in the fusion of endosomal vesicles with the TGN, during which its GTPase activity is strictly regulated by two proteins, Rgp1p and Ric1p, acting in a complex as nucleotide exchange factor of Ypt6p [38]. As illustrated in Figure 2b, fluorescence of *Δypt*6 (104.1% ± 5.0%) and *Δrgp*1 cells (98.1% ± 3.5%) significantly exceeded the defined threshold and resulted in values that closely reflected reporter fluorescence in the negative controls (100.0% ± 3.2%), indicating that Ypt6p and its regulator Rgp1p are involved in RTA transport in yeast. This result nicely matches reports in mammalian cells in which ricin transport from endosomes to the Golgi has also been shown to be regulated by Rab6A and Rab6A′, the human homologues of yeast Ypt6p [5,7].

Since in yeast, Tlg2p and GARP complex components such as Vps51p, Vps52p, Vps53p and Vps54p are known to mediate vesicle docking at the TGN [28], we asked if a loss of any of these components affects RTA transport. Furthermore, Stx16—the mammalian homologue of Tlg2p—has been described to interact with the GARP-complex [39], and Stx18 in conjunction with GARP is required for intracellular ricin trafficking in mammalian cells [5]. As illustrated in Figure 2b, spheroplasts of both a *Δvps*54 (114.5% ± 7.6%) and a *Δvps*51 (101.3% ± 0.8%) knock-out showed a significant increase in GFP fluorescence compared to wild-type, while the corresponding values in *Δvps*52 (52.5% ± 3.1%) and *Δtlg*2 (66.9% ± 2.2%) cells were clearly below the threshold, suggesting that not all GARP complex components are required for RTA transport in yeast. It is widely accepted that GARP-complex stability depends on each subunit and mutations in any component lead to strong missorting and mislocation defects [40]. Unexpectedly, similar results were obtained in mammalian cells in which a siRNA mediated knock-down of the GARP-complex components Vps54 and Vps53, in contrast to Vps52, resulted in a significant decrease in ricin sensitivity [5]. Although the unaffected ricin sensitivity seen after Vps52 knock-down had been interpreted as a false negative result, our analogous observation in yeast indicates that the so far postulated model for GARP-complex assembly and function in the absence of Vps52 might indeed be correct, at least with respect to intracellular RTA transport. Furthermore, and in contrast to the documented role of Syn16 in ricin transport in mammalian cells [5,41], the present result on its homologue in yeast indicates Tlg2p—in contrast to Ypt6p—dispensable for endosome-to-Golgi transport of RTA.

As a further candidate, the impact of the membrane protein Sft2p on RTA transport was studied. In yeast, it has been postulated that Sft2p facilitates fusion of endosome-derived vesicles with the late Golgi. Furthermore, Sft2p genetically interacts with Sed5p, the yeast homologue of mammalian syntaxin 5 (Stx5), and Sft2p overexpression can suppress some *sed5* alleles [42]. Interestingly, mice treated with retro-2 to selectively prevent fusion of Stx5 containing vesicles with the TGN were shown to survive a lethal dose of ricin [6]. We now show that a yeast *Δsft*2 knock-out shows a strong increase in GFP fluorescence (84.1% ± 5.8%) (Figure 2b), indicating that Sft2p is likewise required for efficient retrograde RTA transport in yeast. As the retromer complex is another essential component with a proposed function in retrograde endosome-to-Golgi transport [43], we tested the corresponding yeast proteins Vps5p, Vps17p, Vps35p, Pep8p/Vps26p and Vps29p [44] for their involvement in RTA trafficking. Although siRNA knock-down of several retromer complex components did not affect ricin sensitivity of mammalian cells [5], a yeast *Δsnx*4 knock-out resulted in a dramatic decrease in GFP fluorescence (37% ± 1.8%), while neither *Δpep*8, *Δvps*5, *Δvps*17, *Δvps*29 and *Δvps*35 nor *Δsnx*41 cells significantly differed from wild-type after RTA-treatment (Figure 2b). Both Snx4p and Snx41p, forming a complex in vivo, belong to the family of sorting nexins which, in conjunction with the retromer complex, mediate various retrieval pathways from endosomes to the TGN [45]. Since Snx4p can form a complex with Snx42p/Atg20p (which was not tested here) that also plays a role in endosomal sorting [45], we cannot exclude that the observed absence of a protective effect against RTA in a *Δsnx4* background is due to redundancy. Nevertheless, our data clearly indicate that the tested sorting nexins and retromer complex components are not important for intracellular RTA transport in yeast (Figure 2b), thus nicely matching similar observations in mammalian cells [5]. To analyze proteins involved in COPIB-mediated intra-Golgi transport [46], cells of a *Δgcs*1 and a *Δsec*28 knock-out mutant were tested. While Gcs1p is a regulatory protein in COPIB-transport, Sec28p is a structural COPIB component responsible for complex stability [47,48]. As shown in Figure 2b, reporter fluorescence was not significantly affected in either mutant (*Δgcs*1, 66.3% ± 1.2%; *Δsec*28, 59.6% ± 2.7%), suggesting that COPIB-vesicles are not involved in RTA-transport in yeast.

### 2.4. Impact of Golgi-to-ER Transport and/or Endocytosis Components on RTA Toxicity

Since recent studies in mammalian cells indicated that ERGIC2 (ER/Golgi intermediate compartment) is an important regulator in Golgi-to-ER trafficking of ricin and siRNA-mediated knock-down of ERGIC2 renders cells resistant against high doses of ricin [5], we tested various yeast mutants defective in Golgi-to-ER transport for potential effects on RTA in vivo activity. The yeast homologues of ERGIC2 (Erv41p) and ERGIC3 (Erv46p) form an active complex cycling between the ER and Golgi, which is important for membrane fusion in ER/Golgi transport [49,50]. When analyzed in the yeast assay (Figure 2b), cells of a *Δerv*46 knock-out mutant caused a significant increase (77.5% ± 3.8%) in fluorescence compared to wild-type. Since Erv46p is only functional when present in a complex with Erv41p, the result strongly indicates that the Erv41p/Erv46p complex participates in RTA trafficking and that Golgi-to-ER transport of RTA in yeast shows a striking similarity to ricin transport in mammalian cells. However, as it was recently reported that a direct expression of RTA in the yeast ER lumen requires a cycling between the ER and the Golgi as a pre-requisite for RTA dislocation from the ER to the cytosol [9,35], it cannot be excluded that yeast mutants defective in Golgi-to-ER transport might likewise disrupt the recycling of ER-localized RTA rather than its initial transport to the ER. Since recent studies in yeast also demonstrated that the Erv41p/Erv46p complex serves as retrograde receptor for the retrieval of non-HDEL-bearing ER residents [51], it is conceivable that RTA utilizes this retrieval pathway for retrograde transport from the Golgi to the ER.

To include central regulators of Arf GTPases in the present analysis, Glo3p and Gea1p were likewise examined. While Glo3p is an ADP-ribosylation factor and GTPase activating protein (ArfGAP), which regulates Golgi-ER transport, Gea1p represents a guanine nucleotide exchange factor for ADP-ribosylation factors (ARFs) [52,53]. Nucleotide exchange on ARFs is mediated by Gea1p and this mechanism is essential for in vivo Golgi-to-ER transport [54]. As shown in Figure 2b, spheroplasts of *Δgea*1 (83.5% ± 2.7%) and *Δglo*3 (104.1% ± 1.2%) mutants showed fluorescence values exceeding the threshold of 75% after RTA-treatment, indicating that both proteins are important for RTA trafficking from the Golgi to the ER. Considering that Glo3p and Gea1p are likewise known to be equally important for the regulation of Arf GTPases in yeast, we hypothesize that Arf GTPases might also be involved in retrograde RTA transport.

We next analyzed Sec22p and Rer1p for a potential role in RTA trafficking; Rer1p acts as a retrieval receptor in returning membrane proteins to the ER [55]. In contrast, Sec22p is an R-SNARE present in a complex with Bet1p, Bos1p and Sed5p [56] that constantly cycles between the Golgi and the ER and is responsible for both anterograde and retrograde transport [57]. Since the mammalian homologue of Sec22p, Sec22B, is also important for ricin toxicity [5], we tested the corresponding yeast knock-out mutants and thereby identified a strong increase in reporter fluorescence in *Δsec22* (93.2% ± 5.8%) and *Δrer1* (83.7% ± 0.6%) cells, indicating that Golgi-to-ER transport of RTA depends on the presence of both Sec22p and Rer1p. In support of the proposed function of Ypt6p in the retrograde transport and recycling of Sec22p from the Golgi to the ER [36], we now demonstrate that yeast cells lacking Ypt6p show an RTA-resistant phenotype (Figure 2b), indicating that Ypt6p as Rab GTPase might be involved in regulating endosome-to-TGN as well as TGN-to-ER transport. Furthermore, the data obtained here for Sec22p underlines the similarity in Golgi-to-ER transport of RTA in yeast and ricin trafficking in mammalian cells. For host cell intoxication by the RTA/RTB holotoxin, it has been proposed that RTB binds to KDEL-bearing proteins in the Golgi and thereby hijacks ER residents for retrograde transport to the ER [19,20,58]. Based on the data presented here, we assume the existence of several alternative pathways, including Rer1p, Sec22p and Arf proteins, to ensure efficient RTA transport from the Golgi to the ER. Although Rer1p and the Arf regulators Glo3p and Gea1p have, to our knowledge, not yet been described as important factors in ricin trafficking in mammalian cells, they might be promising novel candidates for ricin transport in higher eukaryotic cells.

To extend the analysis to gene products that potentially affect endocytotic RTA uptake from the plasma membrane, proteins such as Syn8p, Sso1p and Snc1p were selected as candidates for further analysis [59,60,61,62]. As illustrated in Figure 2b, a significant increase in fluorescence in the respective knock-out mutants strongly indicated that Syn8p (82.8% ± 2.6%), Sso1p (78.2% ± 8.6%) and Snc1p (85.4% ± 4.9%) have an impact on RTA trafficking. It is generally accepted that the v-SNARE Snc1p together with Tgl2p is required for both, secretory vesicle trafficking to the plasma membrane and retrograde vesicle transport from early endosomes to the TGN [59,63]. However, in the case of RTA transport, the results obtained here for *Δsnx*4 and *Δsnx*41 mutants do not argue for an involvement of Snc1p in endosome-to-Golgi transport as no increase in GFP fluorescence was observed after RTA-treatment in either mutant and both sorting nexins have been described to be involved in retrieving Snc1p from endosomes to the Golgi [45]. In addition, the significant impact observed in the *Δsso*1 mutant fosters the assumption that Snc1p might indeed be involved in the endocytosis of RTA. It is known that v-SNAREs such as Snc1p confer the docking and fusion of two classes of secretory vesicles by forming a functional SNARE complex with the plasma membrane t-SNAREs Sso1, Sso2, and Sec9 [61,62,64,65]. Thus, yeast cells lacking Sso1p should be blocked in the fusion of Snc1p containing secretory vesicles which, in turn, would negatively affect efficient RTA endocytosis. At least for the depletion of Syn8p, an increased resistance against external applied RTA was observed. Syn8p forms a complex with Snc1p and it is proposed that both Syn8p and Snc1p play a role in plasma membrane-to-endosome transport [60]. It is thus conceivable that a knock-out of *SYN8* disturbs efficient retrograde RTA transport to early endosomes. However, Sso1p, which shows similarities to mammalian Syn1A and Syn1B, as well as the VAMP3 homologue Snc1p are important for RTA trafficking in yeast [66,67]. Furthermore, the yeast homologue of mammalian Syn8, Syn8p, is also part of this transport step. Whether these proteins are also involved in ricin endocytosis in mammalian cells is unknown.

**Figure 3:**
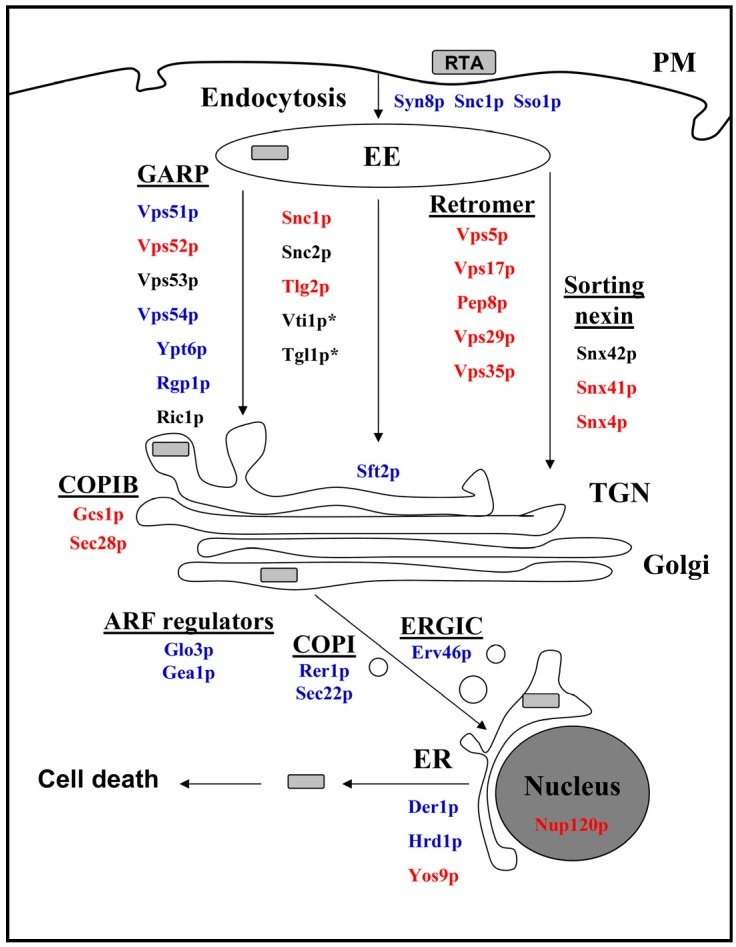
Overview of cellular components involved in RTA uptake and intracellular trafficking in yeast. Proteins involved in RTA transport are shown in blue, non-involved proteins in red; proteins marked with an asterisk (*) are essential for cell viability in yeast; proteins shown in black were not tested. EE, early endosome; PM, plasma membrane; TGN, trans-Golgi network; ER, endoplasmic reticulum; ERGIC, ER/Golgi intermediate compartment; COPI, coat protein I vesicle.

Based on the proteins identified in the present study, we propose a refined model of intracellular RTA transport (Figure 3), which shows striking similarities to ricin holotoxin trafficking in mammalian cells, suggesting yeast as an attractive and powerful model to dissect single steps and pathways of host cell intoxication. We could demonstrate that retrograde transport pathways, including Sft2p and various GARP complex components, regulate intracellular toxin trafficking, while the retromer complex and COPIB vesicles are not part of the transport machinery. Proteins such as Sso1p, Snc1p, Rer1p, Sec22p, Erv46p, Gea1p and Glo3p were identified as novel components of retrograde RTA transport and, therefore, might represent promising novel candidates for future analyses in mammalian cells. Based on the reporter assay developed here, we intend to perform a genome-wide screen to identify additional novel gene products that are required for RTA uptake and subsequent transport through a target cell.

## 3. Materials and Methods

### 3.1. *Escherichia coli* Strains, Plasmids, Culture Media and Genetic Techniques

Standard molecular manipulations were performed as described [68]. *E. coli* TOP10 (*F’mcrA* Δ (*mrr*-*hsdRMS*-*mcrBC*) Φ80*lac-Z*Δ*M15* Δ*lacX*74 *recA*1 *araD*139 Δ (*ara*-*leu*) 7697 *galU galK rpsL* (*StrR*) e*ndA*1 *nupG*) was used for cloning. Construction of the RTA expression plasmid, pET-RTA_His_, was previously described [17]. Vectors pPGK-M28I [32] and pUG35 [69] were used as template to amplify K28_SP_-GFP by SOE-PCR [70] and to obtain the final reporter plasmid pRS315-K28_SP_-GFP. In the first amplification, the sequences of the K28 pptox signal peptide and yeast-enhanced GFP were amplified using HiFi polymerase (Roche) and the following primer pairs: 5′-K28_SS_ (5′-CTCGAGGGATTCATGGACTTCAGTGCTGCTACTTGCGTA-CTGATG) and 3′-K28_SS_-GFP-SOE (3′-CCTCGCCCTTGCTCACACCCCGTGCATATTTGAGATT) for K28_SS_ as well as 5′-K28_SS_-GFP-SOE (5′-AATCTCAAATATGCACGGGGTGTGAGCAAGGGCGAGG) and 3′-GFP (3′-AGATCTAAGCTTGTCGACTTACT TGTACAGCTCGTCCAT) for GFP. In a second step, both fragments were amplified by PCR in the presence of the 5′-K28_SS_ and 3′-GFP primers. The primer introduced a 5′ *Eco*RI and 3′ *Sal*I cleavage site, respectively (underlined). After amplification, the gene fusion was subcloned into pYES2.1/V5-His-TOPO (Invitrogen) and routinely sequenced. The final fusion was cloned as *Eco*RI/*Sal*I fragment under transcriptional control of the *GAL1* promoter in a centromeric yeast expression vector derived from pRS315 [71] to obtain the expression vector pRS315-K28_SP_-GFP.

### 3.2. Affinity Purification of RTA

Purification of RTA was performed as previously described [17]. After purification of (His)_6_-tagged RTA by Ni^2+^/NTA chromatography, eluted protein fractions were desalted and equilibrated in 0.8 M sorbitol. An equally purified cell lysate of *E. coli* expressing the empty vector pET24a^(+)^ without RTA served as negative control. After concentration through 10 kDa cut-off spin columns (Sartorius, Viva Spin 20, Göttingen, Germany), purified proteins were filter sterilized and stored at 4 °C. Total protein content was determined using a BCA protein assay kit (Pierce, Waltham, MA, USA).

### 3.3. Yeast Strains, Transformation and Culture Media

The *S. cerevisiae* wild-type strain BY4742 (MATα *his3*Δ1, *leu2*Δ0, *lys2*Δ0, *ura3*Δ0) and its isogenic knock-out mutants were obtained from Open Biosystems (Lafayette, CO, USA). Yeast cultures were grown at 30 °C, either in YEPD media or in synthetic media containing 2% glucose (SD medium) or 2% raffinose (SR medium). Synthetic medium lacking leucine (SDL or SRL) was used for transformed yeast cells. Yeast transformation with the expression plasmid pRS315-K28_SP_-GFP was achieved by the lithium acetate method [34]. Positive clones were selected on SDL agar. For spheroplast preparation, yeast cells carrying pRS315-K28_SP_-GFP were grown in SRL medium at 30 °C to late exponential phase, harvested at 8000 rpm and washed twice with sterile water. Subsequently, 5 × 10^8^ cells were resuspended in 50 mL spheroplasting buffer (0.8 M sorbitol, 10 mM Tris-HCl (pH 7.5), 10 mM CaCl_2_, 2 mM DTT and 200 µg/mL zymolyase 20 T), incubated at 30 °C for 90 min at 100 rpm, harvested at 4 °C and 2000 rpm, washed twice with 0.8 M sorbitol stabilized SRL medium, and finally resuspended in 5 mL stabilized SRL medium. Subsequently, spheroplasts were used in the GFP-fluorescence assay. To control preparation efficacy, 2 × 10^7^ spheroplasts were centrifuged for 10 min at 2000 rpm after spheroplast preparation, resuspended in H_2_O distilled, shaken for 30 s and plated out on SDL agar for three days at 30 °C. For data evaluation, only samples with efficiency higher than 98% (i.e., less than 2% of non-spheroplasted cells) were used.

### 3.4. GFP-Fluorescence Assay

Yeast cells carrying the expression vector pRS315-K28_SP_-GFP were spheroplasted as described above. Resuspended spheroplasts (2 × 10^7^ cells in 200 µL) were seeded in 96 microtiter plates (Nunc, Roskilde, Denmark). For induction of GFP-expression, 30 µL of 30% galactose solution and 70 µL stabilized SRL medium containing purified RTA or the negative control were added, yielding a final RTA concentration of 5 µM, corresponding to 160 µg/mL RTA. GFP-fluorescence (485 nm/527 nm) was measured every 10 min over 20 h. Each experiment was performed at least three times (*n* = 3) at 30 °C, 120 rpm and a shaking diameter of 1 mm. Measurements were carried out in a fluorescence reader equipped with an integrated shaker (Fluoroskan Ascent, Labsystems, Vantaa, Finland). After 20 h incubation, relative GFP fluorescence in % was calculated for the 20 h time point according to the following equation:

$$\mathrm{fluorescence}=\frac{\left({\mathrm{fluorescence}}_{\mathrm{sample}(20h)}-{\mathrm{fluorescence}}_{\mathrm{sample}(0h)}\right)}{\left({\mathrm{fluorescence}}_{\mathrm{negativecontrol}(20h)}-{\mathrm{fluorescence}}_{\mathrm{negativecontrol}(0h)}\right)}$$
